# Drought Stress Mitigating Morphological, Physiological, Biochemical, and Molecular Responses of Guava (*Psidium guajava* L.) Cultivars

**DOI:** 10.3389/fpls.2022.878616

**Published:** 2022-06-02

**Authors:** Muhammad Usman, Syeda Anum Masood Bokhari, Bilquees Fatima, Bushra Rashid, Faisal Nadeem, Muhammad Bilal Sarwar, Muhammad Shah Nawaz-ul-Rehman, Muhammad Shahid, Chaudhary Muhammad Ayub

**Affiliations:** ^1^Institute of Horticultural Sciences, University of Agriculture, Faisalabad, Pakistan; ^2^Department of Horticulture, Muhammad Nawaz Sharif University of Agriculture, Multan, Pakistan; ^3^Centre of Excellence in Molecular Biology, University of the Punjab, Lahore, Pakistan; ^4^Department of Soil Science, Faculty of Agricultural Sciences, University of the Punjab, Lahore, Pakistan; ^5^Center of Agricultural Biochemistry and Biotechnology, University of Agriculture, Faisalabad, Pakistan; ^6^Department of Biochemistry, University of Agriculture, Faisalabad, Pakistan

**Keywords:** antioxidants, expression sequence tags (ESTs), microarray, pyriform guava, water stress

## Abstract

Guava (*Psidium guajava* L.), a major fruit crop of the sub-tropical region, is facing a production decline due to drought stress. Morphophysiological responses to drought stress and underlying transcriptional regulations in guava are, largely, unknown. This study evaluated the drought stress tolerance of two guava cultivars, *viz*. “Gola” and “Surahi,” at morphological and physiological levels regulated differentially by ESTs (Expressed Sequence Tags). The treatments comprises three moisture regimes, *viz*. T_o_ = 100% (control), T_1_ = 75%, and T_2_ = 50% of field capacity. There was an overall decrease in both morphological and physiological attributes of studied guava cultivars in response to drought stress. Nonetheless, the water use efficiency of the “Surahi” cultivar increased (41.86%) speculating its higher drought tolerance based on enhanced peroxidase (402%) and catalase (170.21%) activities under 50% field capacity (T_2_). Moreover, higher proline and flavonoid contents reinforced drought stress retaliation of the “Surahi” cultivar. The differential expression of a significant number of ESTs in “Surahi” (234) as compared to “Gola” (117) cultivar, somehow, regulated its cellular, biological, and molecular functions to strengthen morphophysiological attributes against drought stress as indicated by the upregulation of ESTs related to peroxidase, sucrose synthase (SUS), alcohol dehydrogenase (ADH), and ubiquitin at morphological, biochemical, and physiological levels. In conclusion, the drought stress acclimation of pear-shaped guava cultivar “Surahi” is due to the increased activities of peroxidase (POD) and catalase (CAT) complimented by the upregulation of related ESTs.

## Introduction

Guava (*Psidium guajava* L.) is a major fruit crop of tropical and sub-tropical regions of the world (Rodríguez et al., 2010). It is rich in nutrients containing flavonoids, dietary fibers, and vitamins A, B, and C (Prakash et al., 2002; Rai et al., 2010). Its leaves and fruit have medicinal value for diarrhea, inflamed mucous membranes, dysentery, sore throat, laryngitis, mouth swelling, anorexia, cholera, skin problems, digestive problems, gastric insufficiency, and ulcers (Alvarez-Suarez et al., 2018). It is native to the American continent and has a very broad center of origin from Mexico to Peru and Brazil (Pereira et al., 2017). Guava grows well from sea level to 2,100 m of altitude; however, for better cultivation, the optimal climatic conditions, including 20–30°C, 1,000–2,000 mm well-distributed annual rainfall, better drainage, and 5–7 pH, are required (Dinesh and Reddy, 2012). It is a commercial crop for many countries such as Pakistan, India, Bangladesh, Brazil, Thailand, and West Indies (Pereira et al., 2017). Annual production of 547,000 tons, from 56,000 hectares, ranks guava as the third most important fruit crop of Pakistan. However, guava production in Pakistan has declined during the past 5 years, predominantly, due to its susceptibility to biotic and abiotic stresses (Usman et al., 2015, 2020; Shah et al., 2019).

Abiotic stresses, resulting from global climate change, severely reduce agricultural production worldwide (Prasch and Sonnewald, 2013; Suzuki et al., 2014; Mahalingam, 2015; Siddiqui et al., 2021; Zulfiqar and Ashraf, 2022). Pakistan stands among the developing countries most affected by global climate change. Drought is the most prevalent of abiotic stresses which seriously threaten sustainable food production through negative regulation of plant growth and development (Bray, 1997; Bartlett et al., 2019; Kogan et al., 2019). Low rainfall and less water availability for irrigation cause water dearth conditions to prevail all over the country, especially in Sindh and Baluchistan provinces (Salma et al., 2012). Over the years, plants have tailored responses to drought stress through physiological, biochemical, molecular, and/or genetic manipulations (Chaves et al., 2003; Izanloo et al., 2008; Xu et al., 2009); however, these responses could be genotype-dependent within a species. In fruit crops, competition may occur between organs for carbohydrates and water resource distribution under stress conditions due to simultaneous growth at vegetative and fruit development levels (Berman and DeJong, 1996). Drought stress, thus, differentially affects the vegetative and reproductive growth of fruit crops (Yuan et al., 2010). For instance, peach and olive decrease shoot growth rate similar to fruit fresh weight when water potential in the stem decreases (Solari et al., 2006; Mirás-Avalos et al., 2016). The changes in gene expression patterns at the transcription level play a crucial role in imparting drought stress tolerance in plants (Lei et al., 2015; Min et al., 2016; Yadav et al., 2018b). Microarray technology has been used to explore the changes in genetic expression during the fruit development of pear, apple, and strawberry (Fonseca et al., 2004; Lee et al., 2007; Moyano et al., 2018). However, the transcriptional bases of morphological, physiological, biochemical, and molecular responses of guava to drought stress have not been reported yet. Extreme events in global climate change are expected to further increase the intensity of drought (Rahmati et al., 2018). Hence, the interpretation of responses and adaptations of guava to drought stress becomes imperative to enhance its drought resilience. This study reports the differential expression of ESTs (Expressed Sequence Tags) in cellular, biological, and molecular processes imparting morphological, physiological, and biochemical alterations in two guava cultivars, “Gola” and “Surahi,” under drought stress conditions.

## Materials and Methods

### Plant Material

This experiment was carried out in the greenhouse of Fruit Plant Nursery Area, Institute of Horticultural Sciences (IHS), University of Agriculture, Faisalabad (UAF), Pakistan. One and a half years old 30 uniform and healthy plants of two white flesh guava cultivars *viz*. “Gola” (round-shaped) and “Surahi” (pear-shaped) were selected in compliance with international, national, and/or institutional guidelines.

The plants were grown in plastic containers having 6 kg of soil material comprised of farmyard manure, sand, and silt (1:1:1). The selected plants of chosen guava cultivars were subjected to three field capacity levels, *viz*. T_o_= 100% field capacity (control), T_1_ = 75% field capacity, and T_2_ = 50% field capacity, with one plant per pot in triplicate, in a Randomized Complete Block Design (RCBD). The plants were subjected to drought stress for 120 days during the summer season (April–July) and data were recorded. During the study period, the average climatic factors were measured as atmospheric temperature (27.2–34.4°C), relative humidity (28.8–59.6%), and day length (8.2–10.4 h). To maintain three-field capacity levels, irrigation intervals ranged from 6 to 25 days according to the environment, temperature, and evapotranspiration rate, and the exact amount of irrigation water was calculated by using the following formula:


$$\begin{array}{l} Total\;mass\;of\;water=Mass\;of\;saturation\;paste-Mass\;of\;oven\;\\ \;drysoil \end{array}$$

The saturation percentage (SP) was calculated as;


$$\begin{array}{lll} Saturation\;percentage\;\left(\%\right) & = & \frac{Total\;mass\;of\;water}{Mass\;of\;oven\;dry\;soil}\;\times 100\\ \;Field\;Capacity\;100\;\left(\%\right) & = & \frac{Saturation\;percentage\;\%}{2} \end{array}$$

The saturation percentage of growing media was 24%; hence, its half, that is, 12% was considered as 100% field capacity. The quantity of water required for 100, 75, and 50% field capacity levels in pots containing 6 kg of soil media were calculated as follows:


$$\begin{array}{l} Water\;required\;for\;100\%\;field\;capacity=\frac{12}{100}\;\times 6=0.72\;L\\ \;=720\;ml\;\\ Water\;required\;for\;75\%\;field\;capacity=\frac{75}{100}\;\times 720\;=\;540\;ml\;\\ Water\;required\;for\;50\%\;field\;capacity=\frac{50}{100}\;\times 720=360\;ml \end{array}$$

After 120 days of drought stress treatment, the leaf samples were collected and plants were irrigated as required for 45 days for the recovery from drought stress, and data were recorded.

### Morphological Parameters

Plant height (cm) was measured twice (before and after stress) using a ruler. The difference between both the readings was calculated as net plant height. The number of leaves was counted before and after stress and the difference between both readings was calculated as the net number of leaves. Leaf area (cm^2^) was calculated after multiplying the length and width of the leaf (before and after stress). The difference was calculated as net leaf area. Four leaves from each replicate were harvested and their fresh weight (g) was taken immediately using a digital weighing balance. The leaves were kept in an oven (Memmert-110, Schwabach, Germany) at 70°C for 72 h for dry weight (g).

### Physiological Parameters

The plants were shifted from the greenhouse (45–47°C) to the growth room (32°C). After 24 h, chlorophyll contents (CC) (μg/g), photosynthesis (A) (μmol CO_2_ m^−2^s^−1^), transpiration (E) (μmol H_2_O m^−2^s^−1^), water use efficiency (WUE), sub-stomatal CO_2_ (Ci) (μmol mol^−1^), stomatal conductance to water vapor (gs) (C, μmol m^−2^s^−1^), and leaf temperature (Tch) (°C) were measured from the third recently matured young leaf from the apex by using portable infrared gas analyzer IRGA (LCi-SD, ADC; Bio-scientific Ltd., UK) in five replicates. All measurements were made during the daytime between 10:00 a.m. and 12:00 O'clock.

### Biochemical Parameters

The activity of Superoxide Dismutase (SOD) (IU/mg of protein) was determined by measuring its ability to prevent the photo-reduction of nitroblue tetrazolium (NBT). Enzyme extract was prepared in potassium phosphate buffer (pH 5), vortexed, and centrifuged. The reaction mixture comprises enzyme extract (100 μl), potassium phosphate buffer (pH 5) (500 μl), methionine (200 μl), triton X (200 μl), NBT (100 μl), and distilled water (100 μl). The mixture was kept under UV light for 15 min and then 100 μl of riboflavin was added. The absorbance of reaction mixture was observed at 560 nm using an ELISA plate (Giannopolitis and Ries, 1977). The amount of enzyme restricting 50% of the NBT photo decline was considered as one unit of SOD. Peroxidase (POD) (IU/mg of protein) activity was measured using the solution of POD reaction comprising 20 mM guaiacol (100 μL), 50 mM phosphate buffer (pH 5) (800 μL), gum solution (0.1 ml), and 40 mM H_2_O_2_ (100 μL). Enzyme extract (100 μl) and reaction mixture (100 μl) were added and absorbance was recorded at 470 nm using ELISA plate (Liu et al., 2009). The catalase (CAT) (IU/mg of protein) activity was measured as the amount of H_2_O_2_ consumed and converted to water H_2_O and oxygen O_2_. Enzyme extract (100 μl), used for SOD determination, was taken and 100 μL of 5.9 mM H_2_O_2_ was added in it. The absorbance was recorded at 240 nm on ELISA plate (Liu et al., 2009). An absorbance change of 0.01 units per min was considered as one unit of POD and CAT. Proline contents (μg/g Fw) were estimated (Bates et al., 1973) from 0.5 g of fresh leaf tissue homogenized in 10 ml of 3% sulfosalicylic acid. Homogenate was filtered through Whatman No. 2 filter paper. Later, the filtrate (2.0 ml) was mixed with 2.0 ml of acid ninhydrin solution (Ninhydrin, 1.25 g) and then dissolved in 6 M orthophosphoric acid (20 ml) and glacial acetic acid (20 ml). The mixture was kept for 60 min on ice bath to cool. Finally, toluene (4.0 ml) was added to the solution and mixed vigorously by passing a continuous stream of air for 1–2 min. The absorbance was taken at 520 nm using spectrophotometer.

Total soluble proteins (TSP) (mg/g) were quantified by adding the Bradford reagent to the enzyme extract prepared for SOD analysis and absorbance was observed at 595 nm (Bradford, 1976) using a spectrophotometer. Total phenolic contents (TPC) (mg/g GAE) were estimated by using the Folin–Ciocalteu reagent (FCR) method (Ainsworth and Gillespie, 2007). The 200-μl F-C reagent was added to 100 μl of tissue extract followed by the addition of 800 μl of 700 mM Na_2_CO_3_ and incubation for 2 h at room temperature. The absorbance was measured at 765 nm using a spectrophotometer. Total flavonoid contents (TFC) (mg/g catechin standard) were determined by the previously described method (Dewanto et al., 2002). In brief, 1 ml extract, containing 0.01 mg/L of dry matter, was added in a 10-ml volumetric flask and then mixed with 5 ml of distilled water followed by the addition of 0.3 ml of 5% NaNO_2_. In two consecutive intervals of 5 min each, 0.6 ml of 10% AlCl_3_ and 2 ml of 1 M NaOH, respectively, were added and the absorbance was taken at 510 nm using spectrophotometer. Antioxidants capacity (DPPH radical scavenging assay) (%), of designated plant crude extract and its polar fractions, was evaluated by assessing their scavenging ability toward 1, 1-diphenyl-2-picrylhydrazyl stable radicals (DPPH) (Queiroz et al., 2009).

### Total RNA Extraction, cDNA Synthesis, Hybridization With Drought-Specific Oligonucleotide Probes, and Microarray Data Analysis

The leaf samples, from drought-stressed (50 and 75% field capacities) and control plants (100% field capacity) of both guava cultivars, were taken in liquid N_2_ and stored at −80°C. Total RNA was extracted, quantified, and purified by using the previously described method (Jaakola et al., 2001). cDNA was synthesized with 5-Aminoallyl-dUTP (AA-dUTP). Before coupling unincorporated aa-dUTP molecules were removed. The coupling of the dyes (Cy3 and Cy5) to the aa-dUTP cDNA was performed as per manufacturer's instructions. Microarray spotted slides with 500 drought-specific ESTs from *Gossypium arboreum* and *Gossypium hirsutum*, showing >70% homology to *Arabidopsis thaliana* (NCBI database), were hybridized with the guava cDNA probe. The standard protocols established by ArrayIt (https://shop.arrayit.com/microarray_tools.aspx) provided by MicroGrid610 by Genomic Solutions® were followed. The detailed procedure was followed as described previously (Ahmed et al., 2020).

Microarray UC4 scanner (Genomic Solutions, USA) was used for scanning the slides. For each sample, separate hybridization, using a single dye, was carried out. The intensities of each sample were measured separately and the images were saved. The preliminary analysis of scanned images was carried out with the help of the microarray image analysis software (GeneTAC Integrator, Genomic Solutions, USA) (Saeed et al., 2003). Data were analyzed using GeneSpring GX software (Agilent Technologies, Santa Clara, CA) and the quality parameters of the extracted data were processed using customized technology created for in-house built experimental procedures. Percentile Shift method and Scaling options were used for the data normalization and to overcome inter-array differences. Normalized intensity values were used for *Hierarchical Clustering* using information of the differentially expressed sequences and keeping 1.5-fold change as the cutoff value.

### Gene Ontology, Functional Annotation, and Characterization

The differentially expressed oligos were used to trace original/parent sequence IDs. For GO functional categorization and annotation, these ESTs were saved in FASTA format and run in Blast2Go Pro software (https://www.blast2go.com/) using Cloudblastx for mapping and annotation (Conesa et al., 2005). The functional categorization was done based on cellular components, biological processes, and molecular functions. The differentially expressed common ESTs, upregulated and downregulated, in both guava cultivars were separately categorized.

### Data Analysis

The data were analyzed using a three-way factorial arrangement. Means were compared through the LSD test at 0.05 level of significance in statistics 8.1.

## Results

### Morphophysiological Attributes as Affected by Drought Stress

The exposure of Guava cultivars “Gola” (round-shaped) and “Surahi” (pear-shaped) to three-field capacity levels (100, 75, and 50%) revealed distinct morphological alterations. The responses of both guava cultivars to all the field capacity levels remained non-significant in terms of plant height, number of leaves, leaf fresh weight, and dry weight (Table 1). However, leaf area was decreased as the field capacity level decreased from 100 to 75% and then to 50% (i.e., increasing drought stress) in “Gola” (−32.75 and −53.42%, respectively) and “Surahi” (−31.10 and −67.16%, respectively) cultivars. Comparatively, the “Surahi” cultivar showed a significantly higher leaf area than the “Gola” cultivar at 75% field capacity level (i.e., 25% drought stress) (Table 1). Taking both cultivars (“Gola” and “Surahi”) together, the mean values of plant height, number of leaves, and leaf area were found to be minimum at the maximum level of drought stress (i.e., 50%). Leaf fresh weight was reduced, significantly, under 75 and 50% field capacity as compared to the 100% level in contrast to leaf dry weight which remained non-significant across the three-field capacity levels (Table 1). These morphological reductions were translated into significant decrements in physiological parameters of guava cultivars in terms of minimal chlorophyll content, photosynthesis, transpiration rate, sub-stomatal CO_2_, and stomatal conductance to water vapor ratio under the highest drought stress level (i.e., 50%). The transpiration rate of both the cultivars was decreased with the increasing drought stress, yet the “Surahi” cultivar showed a minimum transpiration rate (0.334 μmol H_2_O m^−2^s^−1^) as compared to “Gola” (0.543 μmol H_2_O m^−2^s^−1^) which enhanced the WUE of “Surahi” cultivar at maximum drought stress, that is, 50% field capacity (Table 2).

**Table 1:** Net plant growth attributed in guava cultivars under drought stress.

**Parameters**	**Field capacity (%)**	**Cultivars**		**Means**	**Percentage change (Gola)**	**Percentage change (Surahi)**
		**Gola**	**Surahi**			
Plant height (cm)	100	18.58 ± 0.92a	24.80 ± 0.92a	21.69 ± 1.20A	0	0
	75	16.54 ± 0.49a	21.38 ± 1.15a	18.96 ± 1.00B	−10.98	−13.79
	50	13.10 ± 0.58a	18.06 ± 1.43a	15.58 ± 1.10C	−29.49	−27.18
	Means	16.07 ± 0.71B	21.41 ± 0.97A			
Number of leaves	100	13.74 ± 0.73a	17.00 ± 0.95a	15.37 ± 0.78A	0	0
	75	−31.00 ± 0.89a	−26.80 ± 1.24a	−28.90 ± 1.00B	−325.62	−257.65
	50	−49.20 ± 1.66a	−45.00 ± 1.87a	−47.10 ± 1.37C	−458.08	−364.71
	Means	−22.15 ± 7.10B	−18.27 ± 6.99A			
Leaf area (cm^2^)	100	24.00 ± 1.00b	32.03 ± 0.68a	28.01 ± 1.45A	0	0
	75	16.14 ± 0.74c	22.07 ± 0.29b	19.10 ± 1.05B	−32.75	−31.10
	50	11.18 ± 0.70d	10.52 ± 1.25d	10.85 ± 0.68C	−53.42	−67.16
	Means	17.10 ± 1.47B	21.54 ± 2.39A			
Leaf fresh Wt. (g)	100	0.374 ± 0.042a	0.354 ± 0.023a	0.364 ± 0.023A	0	0
	75	0.290 ± 0.034a	0.284 ± 0.011a	0.287 ± 0.017AB	−22.46	−19.77
	50	0.196 ± 0.076a	0.252 ± 0.023a	0.224 ± 0.038B	−47.59	−28.81
	Means	0.287 ± 0.035A	0.297 ± 0.016A			
Leaf dry Wt. (g)	100	0.090 ± 0.024a	0.128 ± 0.034a	0.109 ± 0.021A	0	0
	75	0.088 ± 0.034a	0.092 ± 0.040a	0.090 ± 0.025A	−2.22	−28.13
	50	0.070 ± 0.025a	0.068 ± 0.013a	0.069 ± 0.013A	−22.22	−46.88
	Means	0.083 ± 0.015A	0.096 ± 0.018A			

Values are Means ± SE. Small letters represent comparison among interaction means and capital letters are used for overall means. Different letters indicate significance at P > 0.05. Percentage (%) Change = [(values under 75 or 50% Field Capacity – Value under 100% Field Capacity)/Value under 100% Field Capacity] ^*^ 100.

**Table 2:** Net photosynthetic efficiency in guava cultivars under drought stress.

**Parameters**	**Field capacity (%)**	**Cultivars**		**Means**	**Percentage change (Gola)**	**Percentage change (Surahi)**
		**Gola**	**Surahi**			
Chlorophyll contents CC (μg/g)	100	30.87 ± 0.96a	34.90 ± 3.30a	32.89 ± 1.78A	0	0
	75	24.09 ± 1.54a	27.13 ± 0.94a	25.61 ± 1.06B	−21.96	−22.26
	50	14.21 ± 1.44a	17.99 ± 0.78a	16.10 ± 1.12C	−85.79	−48.45
	Means	23.06 ± 2.51B	26.68 ± 2.65A			
Photosynthesis (μmol CO_2_ m^−2^s^−1^)	100	9.08 ± 0.27a	10.72 ± 0.20a	9.90 ± 0.40A	0	0
	75	7.40 ± 0.25a	8.40 ± 0.13a	7.90 ± 0.26B	−18.50	−21.64
	50	4.41 ± 0.04a	5.18 ± 0.38a	4.79 ± 0.24C	−51.43	−51.68
	Means	6.96 ± 0.69B	8.10 ± 0.81A			
Transpiration rate (μmol H_2_O m^−2^s^−1^)	100	2.183 ± 0.147a	0.959 ± 0.084b	1.571 ± 0.284A	0	0
	75	0.930 ± 0.053b	0.747 ± 0.056bc	0.838 ± 0.054B	−57.4	−22.11
	50	0.543 ± 0.041cd	0.334 ± 0.059d	0.439 ± 0.057C	−75.13	−65.17
	Means	1.219 ± 0.252A	0.680 ± 0.098B			
Water use efficiency	100	4.21 ± 0.39d	11.37 ± 0.51b	7.79 ± 1.63B	0	0
	75	7.79 ± 0.38c	11.41 ± 1.05b	9.60 ± 0.95A	85.04	0.35
	50	5.05 ± 0.64d	16.13 ± 1.36a	10.59 ± 2.57A	19.95	41.86
	Means	5.68 ± 0.59B	12.97 ± 0.95A			
Sub-stomatal CO_2_ (μmol mol^−1^)	100	1420.44 ± 97.36a	1165.33 ± 172.8a	1292.89 ± 105.5A	0	0
	75	1057.78 ± 28.94a	861.52 ± 56.71a	959.65 ± 52.31B	−25.53	−26.07
	50	726.11 ± 108.4a	591.46 ± 41.88a	658.79 ± 60.08C	−48.88	−49.25
	Means	1068.11 ± 109.0A	872.77 ± 98.85B			
Stomatal conductance to water	100	0.100 ± 0.006a	0.071 ± 0.005a	0.085 ± 0.007A	0	0
vapor (C, μmol m^−2^s^−1^)	75	0.071 ± 0.004a	0.039 ± 0.010a	0.055 ± 0.008B	−29	−45.07
	50	0.057 ± 0.001a	0.023 ± 0.007a	0.040 ± 0.008C	−43	−67.61
	Means	0.076 ± 0.007A	0.044 ± 0.008B			
Leaf temperature (°C)	100	19.63 ± 1.32a	16.33 ± 0.62a	17.98 ± 0.98C	0	0
	75	25.30 ± 1.69a	20.98 ± 0.41a	23.14 ± 1.24B	28.88	28.48
	50	32.26 ± 0.78a	27.14 ± 0.62a	29.70 ± 1.23A	64.34	66.20
	Means	25.73 ± 1.94A	21.49 ± 1.59B			

Values are Means ± SE. Small letters represent comparison among interaction means and capital letters are used for overall means. Different letters indicate significance at P > 0.05. Percentage (%) Change = [(value under 75 or 50% Field Capacity – Value under 100% Field Capacity)/Value under 100% Field Capacity] ^*^ 100.

### Enzyme Activities, Protein Biosynthesis, and Antioxidant Activities Under Drought Stress

The antioxidant capacity (DPPH) and superoxide dismutase (SOD) activity were found to be non-significant in both guava cultivars (Table 3). Total soluble proteins decreased as the drought stress increased from 75 to 50% in “Gola” (−11.99 and −27.74%, respectively) and “Surahi” (−11.69 and −22.19%, respectively) with “Gola” cultivar showing higher values of total soluble proteins at all three field capacity levels (Table 3). In the same context, total phenolic contents were the maximum in the “Gola” cultivar (15.14%) under maximum drought stress (50% field capacity) (Table 3). Nevertheless, the peroxidase (POD) and catalase (CAT) activities along with proline and total flavonoids contents were observed to be maximum (402, 170.21, 116.75, and 22.23%, respectively) in the “Surahi” cultivar under 50% field capacity (Table 3). Proline contents and total flavonoid contents were also increased, significantly, in “Gola;” however, the “Surahi” cultivar accumulated maximum contents of both under maximum drought stress levels (Table 3). Altogether, the combined effect of drought stress decreased superoxide dismutase (SOD) activity and total soluble proteins and increased the peroxidase (POD) and catalase (CAT) activities which, somehow, elevated the antioxidant capacity (DPPH) of both guava cultivars (19.89% in “Gola” and 32.14% in “Surahi”) (Table 3).

**Table 3:** Biochemical responses in guava cultivars under drought stress.

**Parameters**	**Field capacity (%)**	**Cultivars**		**Means**	**Percentage change (Gola)**	**Percentage change (Surahi)**
		**Gola**	**Surahi**			
Superoxide dismutase (SOD)	100	6.65 ± 0.06a	8.06 ± 0.04a	7.36 ± 0.32A	0	0
(IU/mg of protein)	75	5.07 ± 0.05a	6.52 ± 0.02a	5.80 ± 0.33B	−23.76	−19.11
	50	3.04 ± 0.03a	4.61 ± 0.10a	3.82 ± 0.35C	−54.29	−42.80
	Means	4.92 ± 0.52B	6.40 ± 0.50A			
Peroxidase (POD) (IU/mg of protein)	100	0.140 ± 0.006e	0.150 ± 0.006e	0.145 ± 0.004C	0	0
	75	0.295 ± 0.009d	0.455 ± 0.007c	0.375 ± 0.036B	110.71	203.33
	50	0.510 ± 0.011b	0.753 ± 0.005a	0.631 ± 0.055A	264.29	402
	Means	0.315 ± 0.054B	0.453 ± 0.087A			
Catalase (CAT) (IU/mg of protein)	100	0.260 ± 0.012e	0.292 ± 0.006e	0.276 ± 0.009C	0	0
	75	0.393 ± 0.015d	0.530 ± 0.012c	0.462 ± 0.032B	51.15	81.51
	50	0.657 ± 0.012b	0.789 ± 0.012a	0.723 ± 0.031A	152.69	170.21
	Means	0.437 ± 0.059B	0.537 ± 0.072A			
Proline contents (PRO) (μg/g Fwt)	100	16.27 ± 0.20e	16.66 ± 0.16e	16.47 ± 0.14C	0	0
	75	20.53 ± 0.52d	24.20 ± 0.37c	22.36 ± 0.87B	26.18	45.26
	50	27.11 ± 0.13b	36.11 ± 0.46a	31.61 ± 2.02A	66.63	116.75
	Means	21.30 ± 1.59B	25.66 ± 2.84A			
Total soluble proteins (TSP) (mg/g)	100	319.67 ± 0.88a	222.33 ± 0.67d	271.00 ± 21.77A	0	0
	75	281.33 ± 0.88b	196.33 ± 0.67e	238.83 ± 19.01B	−11.99	−11.69
	50	231.00 ± 0.58c	173.00 ± 1.15f	202.00 ± 12.98C	−27.74	−22.19
	Means	277.33 ± 12.84A	197.22 ± 7.14B			
Total phenolic contents (TPC) (mg/g GAE)	100	295.00 ± 0.58d	194.00 ± 0.58f	244.50 ± 22.59C	0	0
	75	322.67 ± 1.20b	251.33 ± 0.67e	287.00 ± 15.96B	9.38	29.55
	50	339.67 ± 0.88a	299.67 ± 0.88c	319.67 ± 08.96A	15.14	54.47
	Means	319.11 ± 6.52A	248.33 ± 15.27B			
Total flavonoid contents (TFC)	100	308.33 ± 0.88f	445.33 ± 0.67c	376.83 ± 30.64C	0	0
(mg/g catechin standard)	75	340.33 ± 0.88e	503.00 ± 1.15b	421.67 ± 36.38B	10.38	12.95
	50	375.33 ± 0.88d	544.33 ± 1.20a	459.83 ± 37.80A	21.73	22.23
	Means	341.33 ± 9.68B	497.56 ± 14.36A			
Antioxidants capacity	100	68.67 ± 0.33a	46.67 ± 0.33a	57.67 ± 4.92C	0	0
(DPPH radical scavenging assay) (%)	75	75.33 ± 0.88a	53.67 ± 0.88a	64.50 ± 4.88B	9.70	15
	50	82.33 ± 0.88a	61.67 ± 0.67a	72.00 ± 4.65A	19.89	32.14
	Means	75.44 ± 2.01A	54.00 ± 2.19B			

Values are Means ± SE. Small letters represent comparison among interaction means and capital letters are used for overall means. Different letters indicate significance at P > 0.05. Percentage (%) Change = [(value under 75 or 50% Field Capacity – Value under 100% Field Capacity)/Value under 100% Field Capacity] ^*^ 100.

### Correlation Among Morphological, Physiological, and Biochemical Attributes Under Drought Stress

Correlation analysis of morphological, physiological, and biochemical responses of guava cultivars to drought stress revealed high negative correlation of leaf temperature (Tch) and high positive correlation of chlorophyll content (CC) and photosynthesis (A) with plant height (PH_1_), leaf number (LN_1_), leaf area (LA_1_), leaf fresh weight (LFW_1_), and leaf dry weight (LDW_1_) (Table 4). Interestingly, superoxide dismutase (SOD) exhibited a strong positive correlation with plant height (PH_1_), leaf number (LN_1_), leaf area (LA_1_), leaf fresh weight (LFW_1_), and leaf dry weight (LDW_1_) (Table 5). Similarly, superoxide dismutase (SOD) was also positively correlated to the chlorophyll content (CC) and photosynthesis (A) (Table 6). Moreover, the correlation of total soluble proteins with sub-stomatal CO_2_ (Ci), transpiration (E), and stomatal conductance to water vapor (gs) was also strong and positive (Table 6).

**Table 4:** Correlation matrix of morphological and physiological attributes in guava cultivars under drought stress.

	**Tch**	**Ci**	**E**	**gs**	**A**	**WUE**	**CC**
PH1	−0.910^*^	0.370	0.159	0.037	0.850^*^	0.458	0.845^*^
PH2	−0.452	0.947^**^	0.914^*^	0.984^**^	0.567	−0.762	0.582
LN1	−0.898^*^	0.884^*^	0.757	0.712	0.916^*^	−0.215	0.936^**^
LN2	0.636	−0.764	−0.754	−0.699	−0.624	0.271	−0.670
LA1	−0.942^**^	0.738	0.524	0.517	0.973^**^	−0.067	0.964^**^
LA2	−0.376	0.308	0.004	0.274	0.434	0.163	0.429
LFW1	−0.918^**^	0.886^*^	0.786	0.658	0.908^*^	−0.114	0.939^**^
LFW2	−0.948^**^	0.841^*^	0.639	0.631	0.981^**^	−0.121	0.984^**^
LDW1	−0.862^*^	0.616	0.309	0.416	0.918^**^	0.042	0.894^*^
LDW2	−0.825^*^	0.759	0.590	0.629	0.876^*^	−0.263	0.869^*^

The first values denoted as (1) in each parameter refers to net growth after drought stress and second value (2) refers to change after 4 weeks of recovery (irrigation) and maintained at 100% field capacity. PH1, Plant Height after stress; LN1 and LN2, Number of Leaves; LL1, Leaf Length; LW1, Leaf Width; LA1, Leaf Area; LFW1, Leaf Fresh Weight; LDW1, Leaf Dry Weight; LL:LW1, Leaf Ratio; CC, Chlorophyll Contents; A, Photosynthesis; E, Transpiration; WUE, Water Use Efficiency; Ci, Sub-Stomatal CO_2_; gs, Stomatal Conductance to Water Vapor; Tch, leaf Temperature. Bold values indicate Pearson's correlation coefficient as significant (^*^) or highly significant (^**^) at 5% probability.

**Table 5:** Correlation matrix of morphological and biochemical attributes in guava cultivars under drought stress.

	**SOD**	**POD**	**CAT**	**TFC**	**TPC**	**TSP**	**DPPH**	**PRO**
PH1	0.936^**^	−0.412	−0.471	0.390	−0.976^**^	−0.210	−0.966^**^	−0.404
PH2	0.401	−0.898^*^	−0.893^*^	−0.879^*^	−0.010	0.958^**^	0.186	−0.882^*^
LN1	0.881^*^	−0.860^*^	−0.881^*^	−0.321	−0.676	0.486	−0.526	−0.825^*^
LN2	−0.612	0.677	0.672	0.367	0.427	−0.497	0.294	0.603
LA1	0.948^**^	−0.806	−0.822^*^	−0.116	−0.859^*^	0.248	−0.705	−0.803
LA2	0.401	−0.432	−0.415	−0.092	−0.462	0.082	−0.313	−0.358
LFW1	0.890^*^	−0.798	−0.862^*^	−0.286	−0.601	0.519	−0.524	−0.761
LFW2	0.942^**^	−0.868^*^	−0.897^*^	−0.241	−0.771	0.400	−0.620	−0.848^*^
LDW1	0.883^*^	−0.752	−0.758	−0.055	−0.875^*^	0.136	−0.702	−0.745
LDW2	0.828^*^	−0.833^*^	−0.812^*^	−0.256	−0.753	0.334	−0.547	−0.824^*^

The first value (1) in each parameter refers to net growth after stress and second value refers to change after 4 weeks of recovery (irrigation) and maintained at 100% field capacity. PH1, Plant Height after stress; LN1 and LN2, Number of Leaves; LL1, Leaf Length; LW1, Leaf Width; LA1, Leaf Area; LFW1, Leaf Fresh Weight; LDW1, Leaf Dry Weight; LL:LW1, Leaf Ratio; SOD, Superoxide Dismutase; POD, Peroxidase; CAT, Catalase; PRO, Proline contents; TSP, Total Soluble Proteins; TPC, Ttal Phenolic Contents; TFC, Total Flavonoid Contents; DPPH radical scavenging assay, Antioxidants Capacity. Bold values indicate Pearson's correlation coefficient as significant (^*^) or highly significant (^**^) at 5% probability.

**Table 6:** Correlation matrix of different physiological and biochemical attributes in guava cultivars under drought stress.

	**Tch**	**Ci**	**E**	**Gs**	**A**	**WUE**	**CC**
SOD	−0.997^**^	0.669	0.485	0.363	0.972^**^	0.163	0.977^**^
POD	0.695	−0.949^**^	−0.774	−0.901^*^	−0.817^*^	0.569	−0.811
CAT	0.759	−0.967^**^	−0.798	−0.873^*^	−0.862^*^	0.492	−0.861^*^
TFC	−0.015	−0.685	−0.695	−0.893^*^	−0.145	0.927^**^	−0.149
TPC	0.849^*^	−0.311	−0.079	−0.021	−0.808	−0.416	−0.793
TSP	−0.203	0.826^*^	0.855^*^	0.935^**^	0.322	−0.828^*^	0.343
DPPH	0.789	−0.135	0.034	0.208	−0.689	−0.638	−0.686
Proline	0.684	−0.929^**^	−0.753	−0.878^*^	−0.814^*^	0.589	−0.800

The first values (1) in each parameter refers to net change after stress and second value refers to change after recovery by irrigating to maintain 100% field capacity. CC, Chlorophyll Contents; A, Photosynthesis; E, Transpiration; WUE, Water Use Efficiency; Ci, Sub-Stomatal CO_2_; gs, Stomatal Conductance to Water Vapor; Tch, leaf Temperature; SOD, Superoxide Dismutase; POD, Peroxidase; CAT, Catalase; PRO, Proline contents; TSP, Total Soluble Proteins; TPC, Total Phenolic Contents; TFC, Total Flavonoid Contents; DPPH radical scavenging assay, Antioxidants Capacity. Bold values indicate Pearson's correlation coefficient as significant (^*^) or highly significant (^**^) at 5% probability.

### Principle Component Analysis of Drought Stress Response of Morphophysiological and Biochemical Attributes

**Figure 1:**
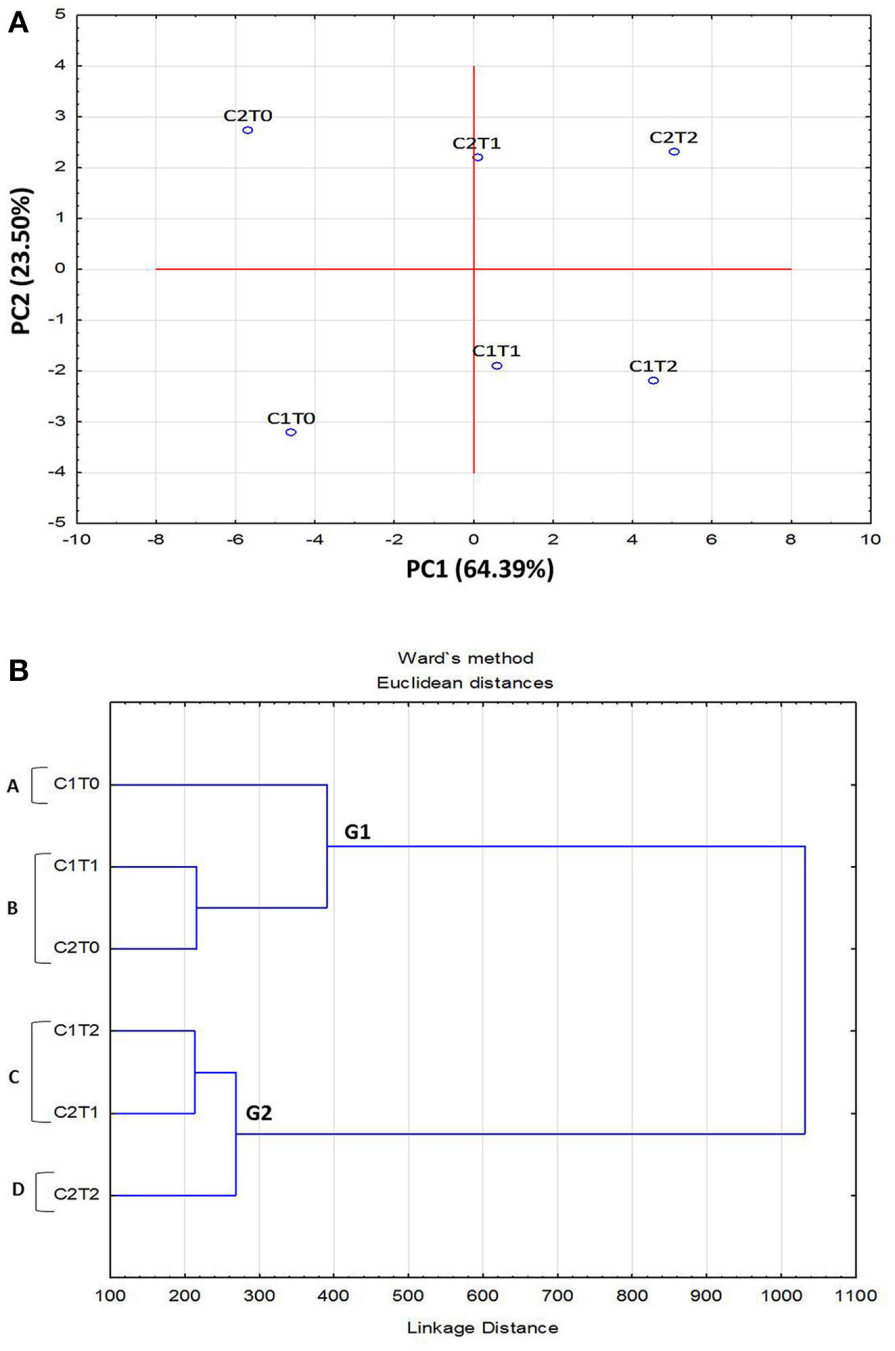
Principal component analysis (PCA) **(A)** and dendrogram **(B)** of morphological, physiological, and biochemical attributes in leaves of guava cultivars under drought stress.

Principle Component Analysis (PCA) of morphological, physiological, and biochemical responses, of “Gola” (C_1_) and “Surahi” (C_2_) cultivars under drought stress, was carried out by classifying them into separate groups. The PCA plot developed was based on the first (PC1) and second (PC2) component factors (64.39 and 23.50%, respectively). Strains C_2_T_o_ and C_1_T_o_, maintained under control conditions, along with C_2_T_2_ and C_1_T_2_, of maximum drought stress, were found to be the most divergent and outliers (Figure 1A). The dendrogram depicted the formation of two main groups (G_1_ and G_2_), where G_2_ grouped higher stress treatments in both cultivars, and four subgroups (A-D) placing “Surahi” (C_2_) with drought stress treatments T_1_ and T_2_ in subgroups C and D (Figure 1B).

### Microarray Analysis and Functional Annotation of Differentially Expressed ESTs in Guava Cultivars Under Drought Stress

**Figure 2:**
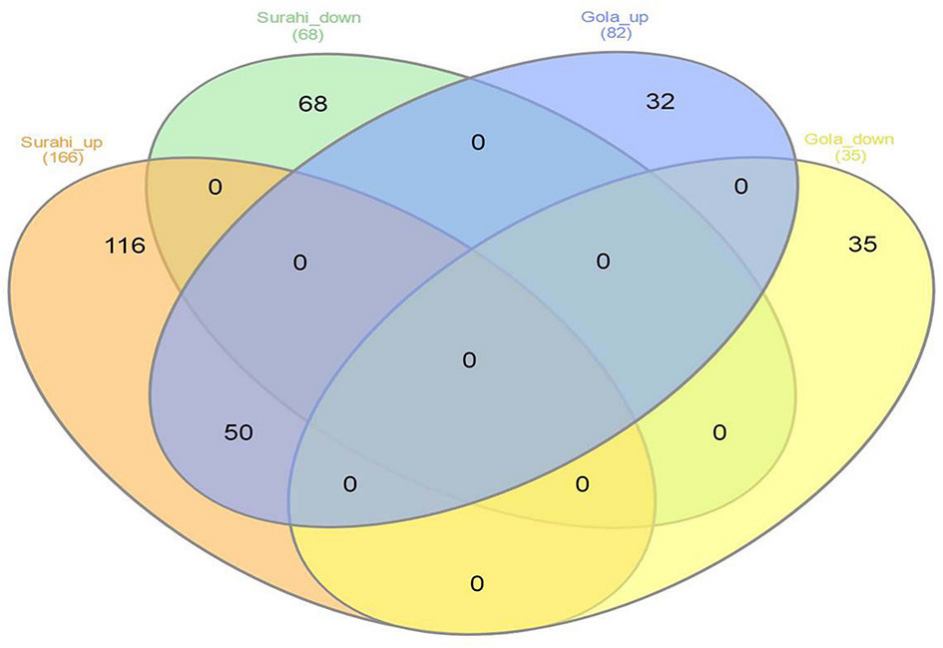
Venn diagram showing differentially expressed ESTs in “Gola” and “Surahi” cultivars of guava under drought stress.

**Figure 3:**
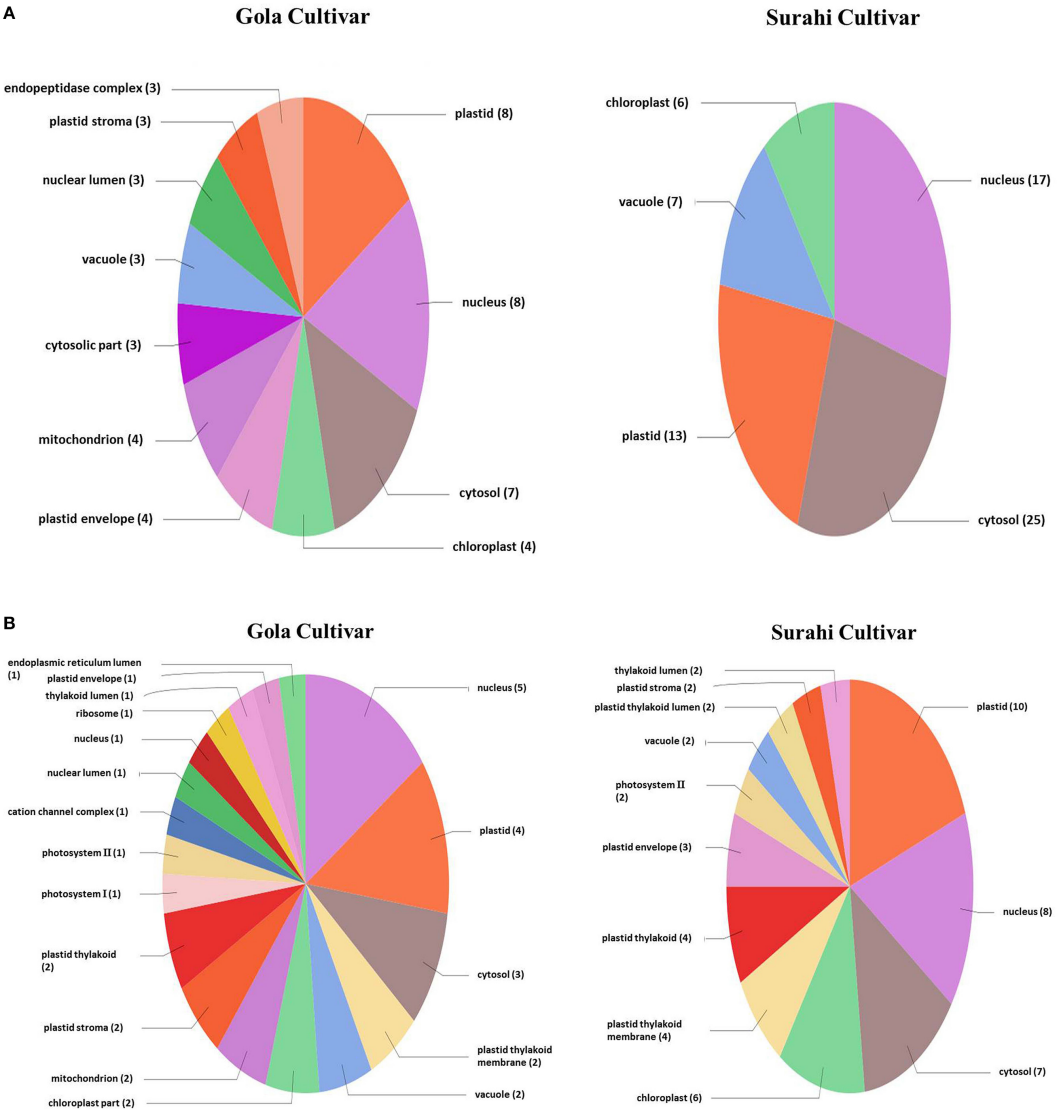
Functional annotation of the upregulated **(A)** and downregulated **(B)** ESTs in cellular components of guava under drought stress.

Microarray analysis underpinned differential expression of 117 ESTs in the “Gola” cultivar, 82 of which were upregulated while 35 were downregulated. Similarly, out of 234 differentially expressed ESTs in the “Surahi” cultivar, 166 were upregulated, whereas 68 were downregulated under drought stress. There were 50 co-upregulated ESTs in “Gola” and “Surahi” cultivars under drought stress (Figure 2). Functional annotation of upregulated ESTs among cellular components of the “Gola” cultivar disclosed 23 sequences belonging to the functions related to the plastid (8), nucleus (8), and cytosol (7). Each chloroplast, plastid envelope, and mitochondrion contained four ESTs individually; whereas, every compartment such as the cytosolic part, vacuole, nuclear lumen, plastid stroma, and endopeptidase complex contained three ESTs each. On the other hand, there were 55 upregulated ESTs belonging to the nucleus (17), cytosol (25), and plastid (13), while 7 belonging to vacuole and 6 to chloroplast of the “Surahi” cultivar in response to drought stress (Figure 3A). Notably, 46% of ESTs, upregulated in cellular components of the “Gola” cultivar, belonged to plastid, nucleus, and cytosol. Similarly, out of the total ESTs upregulated in cellular components of the “Surahi” cultivar, almost 81% of ESTs were upregulated in plastid, nucleus, and cytosol (Figure 3A). Regarding functional annotation of differentially expressed downregulated ESTs, 33 ESTs were downregulated in different cellular components of the “Gola” cultivar under drought stress. Among these, a major component (such as 36%) accounted for the downregulation of five ESTs in nucleus, four in plastid, and three in cytosol. The other 64% cellular component of the “Gola” cultivar contained the downregulated ESTs (Figure 3B). Similarly, 48% of the total downregulated ESTs, in cellular components of the “Surahi” cultivar, belonged to the plastid, nucleus, and cytosol (Figure 3B). The remaining 52% of cellular components contained the other downregulated ESTs (Figure 3B).

**Figure 4:**
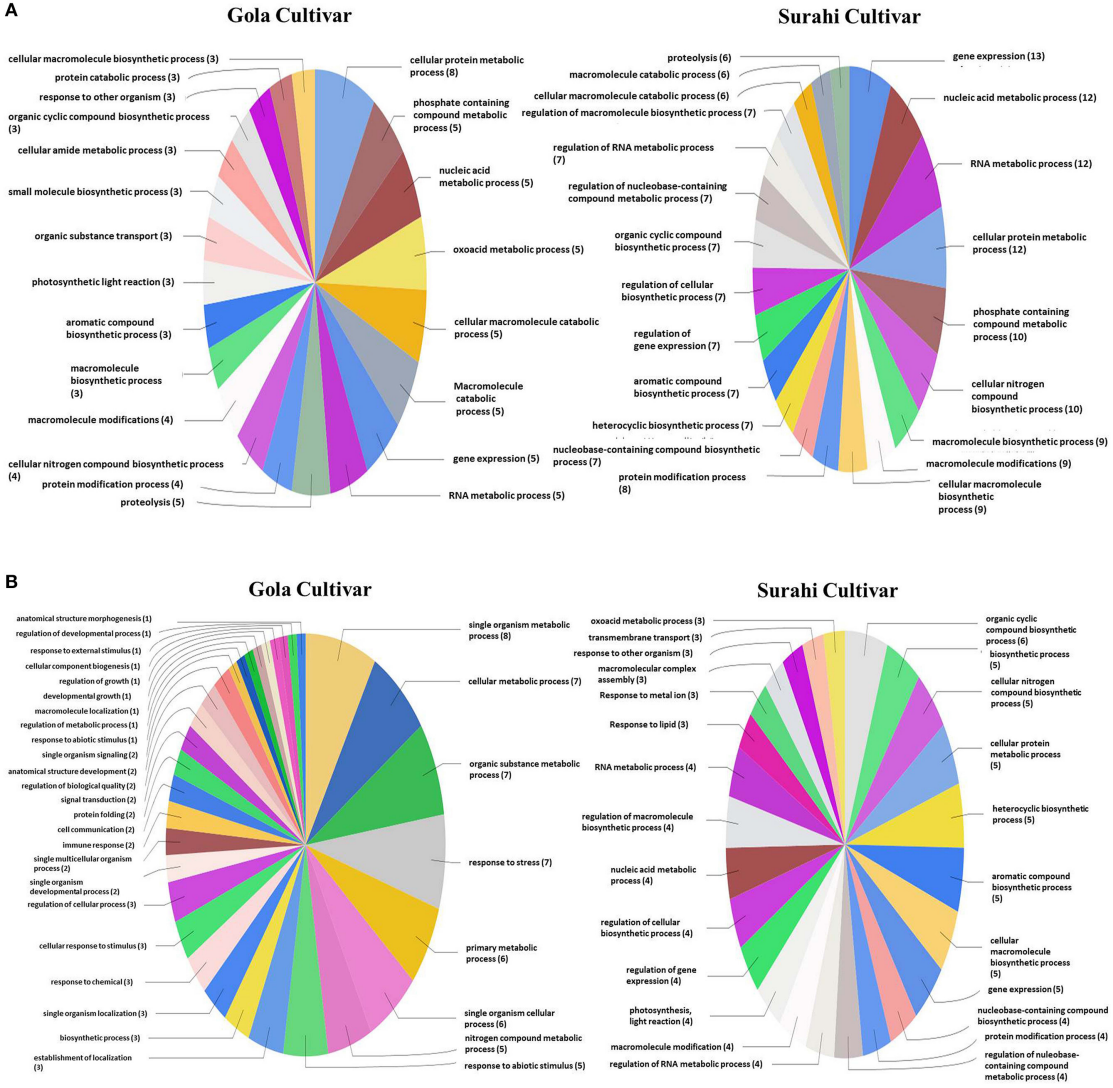
Functional annotation of the upregulated **(A)** and downregulated **(B)** ESTs in biological process of guava under drought stress.

**Figure 5:**
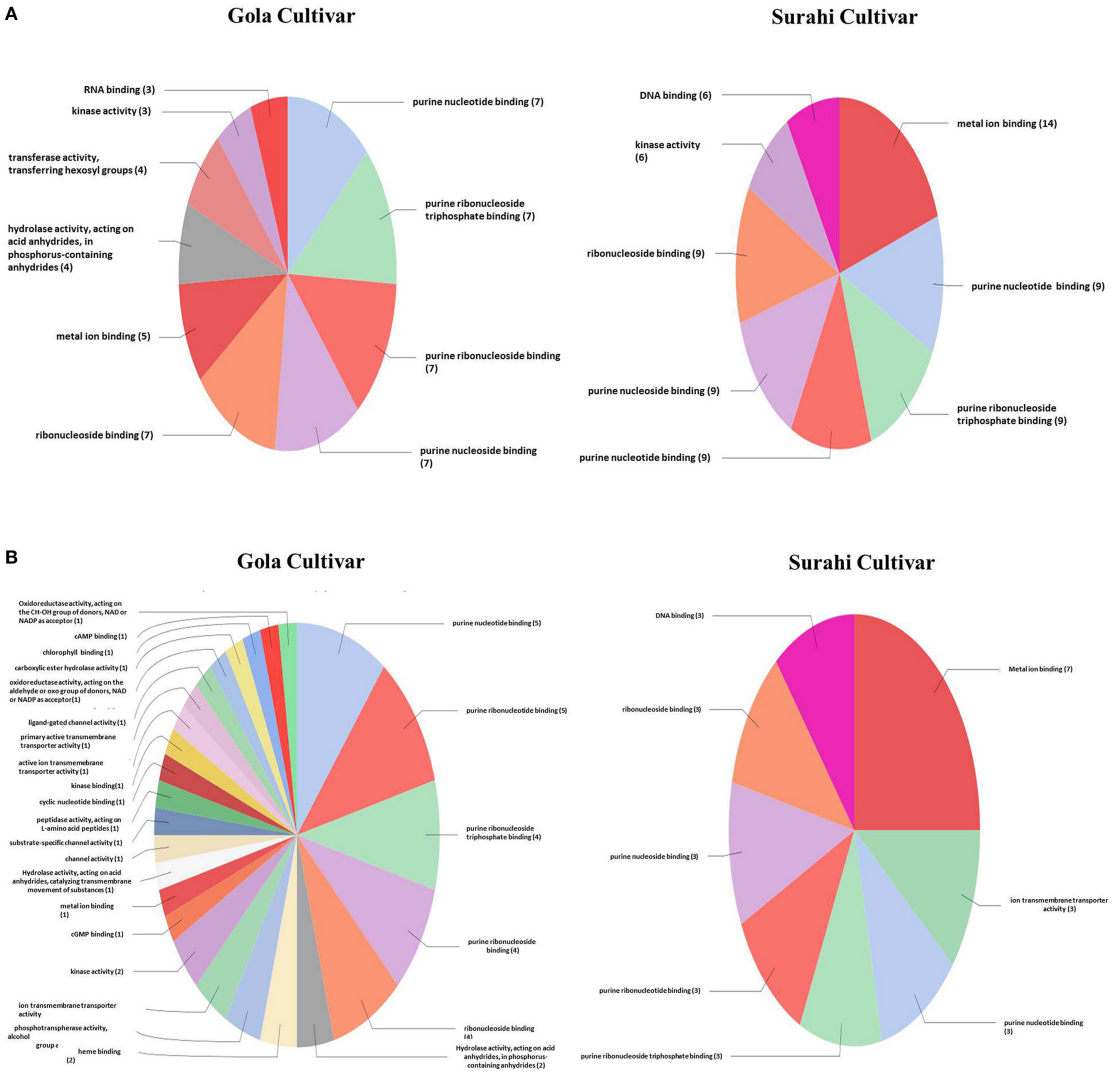
Functional annotation of the upregulated **(A)** and downregulated **(B)** ESTs in molecular function of guava under drought stress.

In response to drought stress, cultivar “Surahi” showed a higher number of ESTs, upregulated (185) and downregulated (103) as compared to the “Gola” cultivar which contained 90 and 98 upregulated and downregulated ESTs, respectively, in most of the biological processes (Figures 4A,B). Among the upregulated ESTs in biological processes of the “Surahi” cultivar, 60 (32.4%) ESTs were involved in various metabolic processes taking place in the nucleus, RNA, cellular protein, phosphate-containing compounds, regulation of RNA, and regulation of nucleo-base RNA compounds. However, comparatively, the “Gola” cultivar expressed 28 (31.1%) upregulated ESTs in the biological processes of various metabolic systems such as cellular proteins, phosphate-containing compounds, nucleic acids, oxoacid, RNA, and cellular amide (Figure 4A). Likewise, 24 (23.3%) and 34 (34.7%) of the total downregulated ESTs in “Surahi” and “Gola” cultivars, respectively, were associated with drought stress-responsive biological processes (Figure 4B). Surprisingly, seven ESTs were observed downregulated, as a result of the stress response, in the biological processes of only the “Gola” cultivar (Figure 4B). A total of 54 and 71 ESTs were upregulated in “Gola” and “Surahi” cultivars, respectively, in the molecular functions resulting from drought stress (Figure 5A). Out of these, five ESTs in the “Gola” cultivar and 14 in “Surahi” were found to coordinate with metal ion binding capacity. Furthermore, there were 36 upregulated ESTs relevant to purine-related molecular functions in “Surahi” as compared to 28 ESTs in the “Gola” cultivar (Figure 5A). Among the downregulated ESTs in molecular functions, 48 belonged to the “Gola” cultivar, whereas only 28 belonged to the “Surahi” cultivar (Figure 5B). Out of 48 downregulated ESTs in the “Gola” cultivar, 18 belonged to purine-related molecular functions, while metal ion binding involved only 1 EST. In contrast, 12 and 7 ESTs were downregulated in molecular functions related to purine binding and metal ion binding, respectively, in the “Surahi” cultivar (Figure 5B).

### Identification of Key Drought Stress Inductive Differentially Expressed ESTs Related to Different Gene Families

Several important ESTs were identified in “Gola” and “Surahi” cultivars in response to drought stress. In addition to similar ESTs upregulated in both cultivars under drought stress (Table 7), ESTs encoding peroxidases (peroxidase-like, thioredoxin-dependent peroxidase 1, and Ascorbate peroxidase 1) and plant regulator RWP-RK family transcription factors were significantly upregulated in “Surahi” as compared to “Gola” cultivar. Sucrose synthase (SUS), alcohol dehydrogenase (ADH), and ubiquitin family genes were also upregulated in the “Surahi” cultivar (Table 8). The drought inducted genes including basic leucine zipper (bZIP) transcription factors were downregulated (Table 9) and putative zinc transporter 11 precursor (ZIP11) was upregulated in the “Surahi” cultivar (Table 8). Ca^2+^/H^+^ exchanger (CAX3) was also downregulated in the “Surahi” cultivar in response to drought stress (Table 9).

**Table 7:** Number of similar ESTs upregulated in “Gola” and “Surahi” cultivars under drought stress.

**Representative gene names**	**Gene IDs (14)**	
	**Gola**	**Surahi**
PSI type III chlorophyll a b-binding	gi|84151489|	gi|189092687|
SAR1 GTP-binding secretory factor	gi|84151425|	gi|84150757|
AC026479_3Strong similarity to alanine aminotransferase from Zea mays gb	gi|84151413|	gi|84150766|
Xyloglucan endotransglucosylase hydrolase 28	gi|84151405|	gi|84150769|
Regulatory particle triple-A ATPase 6A	gi|84151399|	gi|84150782|
scpl20	gi|84151392|	gi|84150796|
Ribosomal S8e family	gi|84151385|	gi|84150798|
Asparagine synthetase (ASN3)(fragment)	gi|84151389|	gi|84150801|
N-terminal nucleophile aminohydrolases (Ntn hydrolases) superfamily	gi|84151383|	gi|84150807|
UDP-Glycosyltransferase superfamily	gi|84151357|	gi|84150814|
Unnamed protein product	gi|84151344|	gi|84150817|
Hypothetical protein AXX17_AT3G01760	gi|84151350|	gi|84150820|
ALPHAVPE	gi|84151332|	gi|84150822|
ASP1	gi|84151319|	gi|84150847|

**Table 8:** Different ESTs upregulated in “Gola” and “Surahi” cultivars under drought stress.

**Representative gene names**	**Gene IDs**	
	**Gola (5)**	**Surahi (44)**
UPL7	gi|84151304|	
Alpha beta-Hydrolases superfamily	gi|84151299|	
RING-H2 zinc finger -	gi|84151291|	
Photosystem II light harvesting complex	gi|84151277|	
Receptor like 4	gi|84151272|	
Hypothetical protein AXX17_AT3G06460		gi|84150852|
RNA-binding (RRM RBD RNP motifs) family		gi|84150854|
Cytochrome c biogenesis precursor		gi|84150856|
SIGB		gi|84150863|
Thioredoxin f1		gi|84150865|
TCP-1 cpn60 chaperonin family		gi|84150883|
Alcohol dehydrogenase		gi|84150892|
Ubiquitin family		gi|84150895|
Calreticulin family		gi|84150898|
ADP-ribosylation factor A1F		gi|84150899|
AC068143_1 an acyl- oxidase from Myxococcus xanthus gb		gi|84150900|
Putative protein		gi|84150960|
Pyruvate kinase family		gi|84150961|
Syntaxin t-SNARE family		gi|84150965|
3–5 -exoribonuclease family		gi|84150975|
Hypothetical protein AXX17_AT4G42150		gi|84150976|
SBP (S-ribonuclease binding) family		gi|84150977|
No pollen germination related 2		gi|84150989|
Dihydroorotate dehydrogenase like		gi|84151092|
RHC1A		gi|84151099|
Alfin-like 7		gi|84151107|
Plant regulator RWP-RK family		gi|84151117|
Unnamed protein product		gi|84151126|
Pyrophosphorylase 1		gi|84151137|
Peroxidase like		gi|84151306|
Thioredoxin-dependent peroxidase 1		gi|84151310|
3-ketoacyl-CoA thiolase, partial		gi|84151321|
ZIP11		gi|84151350|
2C-methyl-D-erythritol 2,4-cyclodiphosphate synthase		gi|84151355|
Alanine aminotransferase		gi|84151389|
Putative protein		gi|84151392|
AF386991_1Unknown protein		gi|84151396|
Ascorbate peroxidase 1		gi|84151398|
Histone H2A		gi|84151415|
AT4G34670		gi|84151420|
Chlorophyll A-B binding family		gi|84151423|
Acyl- N-acyltransferases (NAT) superfamily		gi|84151426|
Putative beta-1,3-glucanase		gi|84151429|
Alpha-helical ferredoxin		gi|84151431|
AF325012_1AT3g47470 (SUS)		gi|84151441|
E3 ubiquitin ligase SCF complex subunit SKP1 ASK1 family		gi|84151442|
AF428301_1At2g28840/F8N16.13		gi|84151459|
Pyridoxal phosphate phosphatase-related		gi|84151480|
Lamin		gi|84151483|

**Table 9:** Number of different ESTs downregulated in “Gola” and “Surahi” cultivars under drought stress.

**Representative gene names**	**Gene IDs**	
	**Gola (13)**	**Surahi (28)**
Chaperone htpG family	gi|194346554|	
Glyceraldehyde-3-phosphate dehydrogenase	gi|194346556|	
Peroxidase ATPA2	gi|189092560|	
Hypothetical protein	gi|189092499|	
SOS3-interacting 3	gi|189092494|	
Cysteine ase	gi|189092488|	
Hypothetical protein AXX17_AT4G12560	gi|189092485|	
60s ribosomal L34	gi|189092479|	
Ketol-acid reductoisomerase	gi|189092476|	
Alcohol dehydrogenase	gi|189092473|	
Photosystem II type I chlorophyll a b binding	gi|189092469|	
Putative protein	gi|189092461|	
Peroxidase ATP2a	gi|189092460|	
R- L3 B		gi|84151074|
Basic-leucine zipper (bZIP) transcription factor family		gi|84151069|
C4-dicarboxylate transporter malic acid transport		gi|84151067|
AGL24		gi|84151065|
CAX3		gi|84151061|
Adenine nucleotide translocase		gi|84151044|
Photosystem II reaction center W		gi|84151053|
PS2		gi|84151030|
AC068143_1 an acyl- oxidase from Myxococcus xanthus gb		gi|84151021|
Growth-regulating factor 2		gi|84151022|
Hypothetical protein AXX17_AT1G23540		gi|84151008|
Calmodulin 7		gi|84151005|
Cytochrome family subfamily polypeptide 6		gi|84151006|
AT1G26850		gi|84151000|
Cysteine ase AALP		gi|84150992|
AF370474_1chlorophyll a b-binding CP29		gi|84150990|
Histone H4		gi|84150959|
Photosystem II subunit P-1		gi|84150967|
M-type thioredoxin		gi|84150951|
Glycosyltransferase family 61		gi|84150948|
Calmodulin 6		gi|84150941|
Voltage dependent anion channel 1		gi|84150938|
2-oxoglutarate (2OG) and Fe(II)-dependent oxygenase superfamily		gi|84150937|
Photosystem I reaction center subunit PSI- PSI- (PSAN)		gi|84150931|
Peroxidase prxr1		gi|84150928|
Glycolate oxidase		gi|84150922|
AT3g53990 F5K20_290		gi|84150920|
RELA SPOT homolog 3		gi|84150909|

## Discussion

Plants respond to drought stress differentially at morphological, physiological, biochemical, and molecular levels (Pigliucci, 2005; Wang et al., 2017). Previous studies have reported the influence of drought stress on fruit quality and size of a peach (Mirás-Avalos et al., 2016; Rahmati et al., 2018), and biochemical responses, cellular ultrastructure, and tree architecture of apple (Šircelj et al., 2005; Wang et al., 2012; Yang et al., 2016). Similar studies are also available involving olive (Fernandes et al., 2018), banana (Muthusamy et al., 2016), cherry (Sivritepe et al., 2008), and wild jujube (Yadav et al., 2018b). However, the response of guava to drought stress has not been widely studied to date.

The morphological alterations, that plants undergo in response to drought stress, involve a decline in growth (Hund et al., 2009). In this study, guava cultivars “Gola” and “Surahi,” individually remained unaffected by drought stress in terms of plant height, number of leaves, leaf fresh weight, and dry weight. However, both cultivars decreased their leaf area under drought stress (75 and 50% field capacity level). Interestingly, the “Surahi” cultivar showed a higher leaf area as compared to the “Gola” cultivar at a 75% field capacity level (Table 1) which indicated its apparent plastic response to water deficiency (Pigliucci, 2005; Wang et al., 2017). To our surprise, the WUE of the “Surahi” cultivar was found to be greater than the “Gola” cultivar at both 75 and 50% field capacity levels, perhaps, owing to its lower values of transpiration rate and unchanged photosynthesis (Table 2) (Hatfield and Dold, 2019). In addition, the enhanced WUE of the “Surahi” cultivar (Table 2) could also be a physiological response resulting from decreased leaf area (Table 1) allowing minimum surface area for leaf water to evaporate (Tátrai et al., 2016).

Adverse environmental conditions, such as drought, can lead to the accumulation of superoxide radicals (${\text{O}}_{2}^{-}$), hydrogen peroxide (H_2_O_2_), and hydroxyl radical (OH). These free radicals and reactive oxygen species (ROS) induce oxidative damage depending upon the range of sensitivity shown by plant species (Rampino et al., 2006; Li et al., 2009; Zulfiqar and Ashraf, 2021). In this study, the activity of superoxide dismutase (SOD) remained non-significant between both guava cultivars; however, the activities of peroxidase (POD) and catalase (CAT), along with proline content were maximized in the “Surahi” cultivar under maximum drought stress (50% field capacity) (Table 3). These enhanced activities of the antioxidant enzymes could have acted as the first line of defense against the negative effects of the oxidative damage in the “Surahi” cultivar (Lee et al., 2007; Sarker and Oba, 2018), as is the case in rice and maize (Nyathi and Baker, 2006; Siddiqui et al., 2021). Obvious differences existed between guava cultivars and treatments where “Surahi” (C_2_) out lied “Gola” (C_1_) in drought stress conditions (Figure 1A), which was also manifested by the dendrogram having “Surahi” (C_2_) and maximum drought stress (T_2_) in subgroup D (Figure 1B).

Microarray analysis provides global changes in gene expression of plants subjected to various environmental stimuli (Sewelam et al., 2014, 2020; Moyano et al., 2018). The microarray analysis distinguished 234 differentially expressed ESTs in the “Surahi” cultivar which was almost double than the 117 differentially expressed ESTs in the “Gola” cultivar under drought stress (Figure 2). This differential expression of 234 ESTs suggested their probable role in pathways related to the drought stress response of the “Surahi” cultivar (Li et al., 2016). The differential expression of ESTs was further analyzed in cellular components, biological processes, and molecular functions of both the guava cultivars to understand their specific contribution toward drought stress tolerance. The upregulation of 55 ESTs was observed in nucleus, cytosol, and plastid of the “Surahi” cultivar in comparison to only 23 upregulated ESTs in the “Gola” cultivar (Figure 3A). Similarly, 25 ESTs were downregulated in nucleus, cytosol, and plastid of “Surahi” than the “Gola” cultivar, where 12 ESTs were downregulated (Figure 3B). The differential expression of a higher number of ESTs in nucleus, cytosol, and plastid of the “Surahi” cultivar might have regulated the cellular network through signal transduction pathways of drought stress tolerance (Luhua et al., 2008, 2013). The differential expression of ESTs in the biological processes of guava cultivars revealed upregulation of 60 ESTs, and downregulation of 24 ESTs in metabolism and stress-related processes, respectively, in the “Surahi” cultivar under drought stress (Figures 4A,B). These numbers are significant as compared to 28 upregulated and 34 downregulated ESTs of “Gola” cultivar in metabolism and stress-related processes (Figures 4A,B). The increased number of upregulated and decreased number of downregulated ESTs in metabolism and stress-related processes elicited the possible alterations of “Surahi” cultivar metabolism to activate protective mechanism against oxidative damage due to drought stress (Rizhsky et al., 2002). Plants, being sessile, respond to various stress conditions through typical signaling cascades at molecular level (Dutta et al., 2018). The stress adaptability of “Surahi” cultivar was also reinforced by the upregulation of 14 ESTs related to metal ion binding in comparison to only 5 ESTs of similar molecular function in “Gola” cultivar (Figure 5A). A possible connection exists between purine catabolism and stress signaling in plants (Watanabe et al., 2014). The downregulation of lesser purine-related ESTs in “Surahi” cultivar (12 ESTs) than “Gola” cultivar (18 ESTs) further elaborated its possible purine metabolite biosynthesis-based drought stress mitigation strategy which can be further explored in future.

Peroxidases regulate cell wall loosening and lignification along with biotic and abiotic stress responses (Yan et al., 2019). The oxidoreduction, between H_2_O_2_ and various reductants, is catalyzed by peroxidases (Hiraga et al., 2001). Peroxidase family gene cysteine protease was involved in ROS detoxification in *Ziziphus nummularia* (Yadav et al., 2018b). Accumulation of cysteine protease (CP) mRNA is also reported in Arabidopsis under drought stress (Koizumi et al., 1993) and tomato under cold stress (Schaffer and Fischer, 1988). ESTs encoding for peroxidases (peroxidase-like, thioredoxin-dependent peroxidase 1, and Ascorbate peroxidase 1) were significantly upregulated in “Surahi” as compared to the “Gola” cultivar (Table 7). This upregulation, correspondingly, enhanced the peroxidase activity of the “Surahi” cultivar, thus facilitating it to cope with the formation of reactive oxygen molecules under drought stress (Reddy et al., 2004), similar to pepper (Sziderics et al., 2010) and wild jujube (Yadav et al., 2018a). Several members of the RWP-RK family transcription factors, such as NLP7, are involved in drought stress tolerance in plants (Castaings et al., 2009). Interestingly, RWP-RK family transcription factors were also significantly upregulated in the “Surahi” cultivar (Table 7). Moreover, the upregulation of Sucrose synthase (SUS) (Table 7), a glycosyltransferase enzyme, proposed its role in sugar metabolism (Stein and Granot, 2019) of the “Surahi” cultivar subjected to drought stress. The sugar products, glucose and fructose, of SUS3 and SUS4 genes were significantly increased in drought-stressed leaves of sweet orange (Goncalves et al., 2019). These sugars play an important role as osmoprotectants, helping to stabilize cell membranes and maintaining cell turgor (Valluru and Van den Ende, 2011). In addition, the upregulation of alcohol dehydrogenase (ADH) could have regulated growth and development, adaptation to stress, fruit ripening, and aroma production (Jin et al., 2016) of “Surahi” cultivar under drought stress (Table 7). Ubiquitin family genes have proteolytic and non-proteolytic roles in response to different environmental clues (Miricescu et al., 2018). An upregulation of ubiquitin family genes was also observed in “Surahi” cultivar (Table 7) building up to aforementioned stress-regulating key players under drought stress. Basic leucine zipper (bZIPs) transcription factors govern many developmental and physiological processes, *viz*. photomorphogenesis, leaf and seed formation, energy homeostasis, and abiotic and biotic stress responses (Corrêa et al., 2008). The drought-induced basic leucine zipper (bZIP) transcription factors (Rodriguez-Uribe and O'Connell, 2006) were downregulated in the “Surahi” cultivar (Table 8) in response to drought stress contrary to what was observed in *Arachis duranensis*. The reason could be the gradual drought stress induction in *Arachis duranensis* (Guimarães et al., 2012) and keeping the control at 70% drought stress, whereas, in our study, control was kept at 100% field capacity level, and 75 and 50% field capacity levels were considered as drought treatments throughout the experiment. Finally, Ca^2+^/H^+^ exchanger (CAX3) was downregulated in “Surahi” cultivar in response to drought stress as reported previously in wild *Arachis magna* and *Vigna radiata* (Brasileiro et al., 2015).

Considering the combined effect of drought stress on “Gola” and “Surahi” cultivars at morphological, physiological, and biochemical levels, the high positive correlation of chlorophyll contents (CC) and photosynthesis (A) with plant height (PH_1_), leaf number (LN_1_), leaf area (LA_1_), leaf fresh weight (LFW_1_), and leaf dry weight (LDW_1_) (Table 4) advocated that despite drought stress, the guava cultivars were being facilitated internally for growth and development at morphological and physiological levels. Moreover, the positive correlations of sub-stomatal CO_2_ (Ci), transpiration (E), and stomatal conductance to water vapor (gs) with total soluble proteins (Table 6) highlighted the biosynthesis of proteins through efficient photosynthetic machinery in guava cultivars under drought stress (Johnson and Stepien, 2016; Sela et al., 2020). Parallel to this, on one hand, superoxide dismutase (SOD) was positively correlated with plant height (PH_1_), leaf number (LN_1_), leaf area (LA_1_), leaf fresh weight (LFW_1_), and leaf dry weight (LDW_1_) (Table 5), while, on the other hand, it also exhibited a strong positive correlation with chlorophyll contents (CC) and photosynthesis (A) (Table 6). These findings, somehow, proved the beneficial antioxidant activities of ROS scavenging enzymes rendering morphological and physiological enhancements in guava cultivars (Pernollet et al., 1986).

## Conclusion

The drought-induced transcriptional regulations of stress tolerance in guava remained unknown to date. This study observed morphological and physiological decreases in white flesh guava cultivars, round-shaped “Gola” and pear-shaped “Surahi,” under drought stress. The increase in leaf area and water use efficiency (WUE) of the “Surahi” cultivar suggested its higher drought tolerance which was also confirmed by increased activities of peroxidase (POD) and catalase (CAT). Furthermore, higher content of proline and total flavonoids reinforced the drought stress retaliation of the “Surahi” cultivar. Microarray analysis revealed differential expression of 234 ESTs in “Surahi” as compared to 117 ESTs in the “Gola” cultivar which indicated the involvement of a larger set of ESTs in cellular, biological, and molecular functions to regulate drought stress withstanding mechanism of “Surahi” cultivar. Finally, upregulation of ESTs related to peroxidase, sucrose synthase (SUS), alcohol dehydrogenase (ADH), and ubiquitin enhanced the cellular, biological, and molecular processes of the “Surahi” cultivar leading to improvements in morphological and physiological functioning under drought stress. These findings provide a useful basic reference to further validate the drought stress inductive candidate genes and explore their functions for the improvements in breeding programs of guava cultivars.

## Data Availability Statement

The original contributions presented in the study are included in the article/supplementary materials, further inquiries can be directed to the corresponding authors.

## Author Contributions

MU and BR designed the main project and experimental plans, main wet-lab experimental work has been done by SB, BF, and MN-u-R. Statistical analyses are performed by FN. Microarray experiments are performed by MBS and BR. The biochemical and physiological data/analyses have been performed and documented by MS and CA. The draft article has been written by MU and edited and revised by BR. All authors read and approved the final version of the manuscript.

## Funding

This research was partially funded by the project # CS 121 sponsored under the Agriculture Linkage Program (ALP) of the Pakistan Agriculture Research Council (PARC), Islamabad, Pakistan.

## Conflict of Interest

The authors declare that the research was conducted in the absence of any commercial or financial relationships that could be construed as a potential conflict of interest.

## Publisher's Note

All claims expressed in this article are solely those of the authors and do not necessarily represent those of their affiliated organizations, or those of the publisher, the editors and the reviewers. Any product that may be evaluated in this article, or claim that may be made by its manufacturer, is not guaranteed or endorsed by the publisher.
